# A review of patient and carer participation and the use of qualitative research in the development of core outcome sets

**DOI:** 10.1371/journal.pone.0172937

**Published:** 2017-03-16

**Authors:** Janet E. Jones, Laura L. Jones, Thomas J. H. Keeley, Melanie J. Calvert, Jonathan Mathers

**Affiliations:** 1 Institute of Applied Health Research, College of Medical and Dental Sciences, University of Birmingham, Birmingham, United Kingdom; 2 Parexel International, Evergreen Building, London, United Kingdom; University of Liverpool, UNITED KINGDOM

## Abstract

**Background:**

To be meaningful, a core outcome set (COS) should be relevant to all stakeholders including patients and carers. This review aimed to explore the methods by which patients and carers have been included as participants in COS development exercises and, in particular, the use and reporting of qualitative methods.

**Methods:**

In August 2015, a search of the Core Outcomes Measures in Effectiveness Trials (COMET) database was undertaken to identify papers involving patients and carers in COS development. Data were extracted to identify the data collection methods used in COS development, the number of health professionals, patients and carers participating in these, and the reported details of qualitative research undertaken.

**Results:**

Fifty-nine papers reporting patient and carer participation were included in the review, ten of which reported using qualitative methods. Although patients and carers participated in outcome elicitation for inclusion in COS processes, health professionals tended to dominate the prioritisation exercises. Of the ten qualitative papers, only three were reported as a clear pre-designed part of a COS process. Qualitative data were collected using interviews, focus groups or a combination of these. None of the qualitative papers reported an underpinning methodological framework and details regarding data saturation, reflexivity and resource use associated with data collection were often poorly reported. Five papers reported difficulty in achieving a diverse sample of participants and two reported that a large and varied range of outcomes were often identified by participants making subsequent rating and ranking difficult.

**Conclusions:**

Consideration of the best way to include patients and carers throughout the COS development process is needed. Additionally, further work is required to assess the potential role of qualitative methods in COS, to explore the knowledge produced by different qualitative data collection methods, and to evaluate the time and resources required to incorporate qualitative methods into COS development.

## Introduction

Clinical trials in health care provide important evidence of the efficacy and safety of interventions and treatments [1], thereby informing future patient care, clinical guidelines and health policy [2, 3]. The selection of outcomes to be measured and reported is an important part of the trial design process. Historically, the selection of outcomes has usually been based on the views of individual study teams informed by clinical and statistical considerations, and guided by regulatory considerations [4–6]. This is problematic since outcomes that matter to key stakeholders, including patients and carers, may be omitted. Furthermore, a wide variety of outcomes may be measured and reported across trials in the same health area “making it difficult or impossible to synthesise the results of different studies” [6] (p1). The difference in outcomes used across studies can also make it hard to detect reporting bias, where authors fail to report all findings because of the desire to report only positive results [7]. Greater uniformity in the reporting of outcomes and measures within a research area would help to facilitate the comparison and synthesis of research findings [7, 8].

One potential solution to this problem is the use of core outcome sets (COS). A COS is an agreed standardised set of outcomes to be measured and reported as a minimum in all trials in a specific health related area [6]. If implemented, a COS may help to ensure that outcomes are relevant to a range of stakeholders and will provide consistent trial outcome data to inform evidence synthesis, clinical practice, shared-decision making and health policy [9, 10]. Stakeholders can include patients, carers, patient representatives and patient advocates (reported as patients and carers from this point onwards), as well as healthcare professionals and decision makers including: funders, researchers, statisticians, health economists and pharmaceutical company representatives (reported as health professionals from this point onwards) [4, 10]. Ultimately, it is the patients and those around them (carers) who benefit from improved healthcare and so it is important that their views and preferences are heard, particularly as there is evidence to suggest that patients’ perspectives may differ from those of clinicians [11].

Despite the potential benefits of including a wide range of stakeholders in COS development, evidence to-date demonstrates that relatively few (18%) COS exercises include patients and carers and the reporting of the process does not always make it clear how they have participated [6]. Gargon et al. [6] recommended that further work is carried out to identify effective methods of eliciting patient and carer views in outcome set development. Data generated using qualitative methods can help to provide in-depth insight into patients’ and carers’ perspectives [12]. Therefore, qualitative methods could be well placed to identify outcomes that are important to patients and carers and to understand why that is. The aim of this review was therefore to: 1) review the methods by which patients and carers have been included as participants in COS development and 2) explore and describe the reported use of qualitative research with patients and carers. For the purpose of this review we were interested in participation, that is where patients and carers contribute data to COS development exercises as research participants, rather than involvement, where they contribute to the research process as an active research partner or advisor.

## Methods

### Data source

The Core Outcome Measures in Effectiveness Trials (COMET) Initiative “aims to bring together people interested in the development, reporting and promotion of COS, derived using rigorous consensus methods” [4]. The COMET Initiative database is an international repository of studies relevant to the development of COS, planned, ongoing and completed [13]. The COMET database was developed based on a systematic review using extensive searches [4] and the COMET Initiative conduct an annual search of the literature in order to keep the database up to date [13]. In addition, planned and ongoing COS exercises can be submitted to COMET by individuals or groups for inclusion in the database [13].

### Search strategy

Given that the COMET database is a comprehensive source for COS development studies with the contents regularly updated, we limited our search to this one database.

#### COS Studies involving patient/carer involvement

The COMET database was searched on the 13 August 2015 using the following search categories: Carer organisations / Support group representatives, Charities, Conference participants, Consumers (caregivers), Consumers (patients), Families, Individuals with a known interest, Patient / Support group representatives, Service users, Guideline developers, with a study type of COS. Inclusion criteria were: papers developing COS with patients and carers as research participants. In addition, reference searches of the included papers were conducted.

#### Qualitative studies to inform COS development involving patients and carers

From these searches we identified all papers that described research using qualitative data collection methods (e.g. focus groups, interviews).

### Data extraction and reporting

A data extraction pro-forma was developed, piloted and used to record study specific information: title, author, year, location of study (country), health area and data collection dates. For COS papers not using qualitative data collection methods we extracted the data collection methods used and the number of health professionals and patient and carer representatives participating in these. For papers reporting qualitative data collection methods we extracted; the stated qualitative methodological framework and rationale for this (please note: in qualitative research the methodological framework guiding research conduct, such as grounded theory, phenomenology or ethnography is distinguished from the methods used during conduct e.g. sampling, data collection, analytic approach) [14]; methods (sampling approach; data collection and analysis); sample characteristics; resource use (costs, resources and time involved); stated strengths and limitations and stated impact of the qualitative research). Some data items were informed by the CASP Qualitative Checklist [15].

The lead author (JJ) extracted data from all included papers and a second researcher (JMM, LLJ, MJC or TJHK) checked the extraction for accuracy on all papers reporting the use of qualitative methods. Any discrepancies were resolved via discussion within the research team. Data extraction were combined when a COS development exercise was reported across more than one paper. Data have been summarised descriptively. Recommendations have been made for the transparent reporting of qualitative research to inform COS development.

## Results

### COS studies with patients and carers

#### Included papers

**Fig 1:**
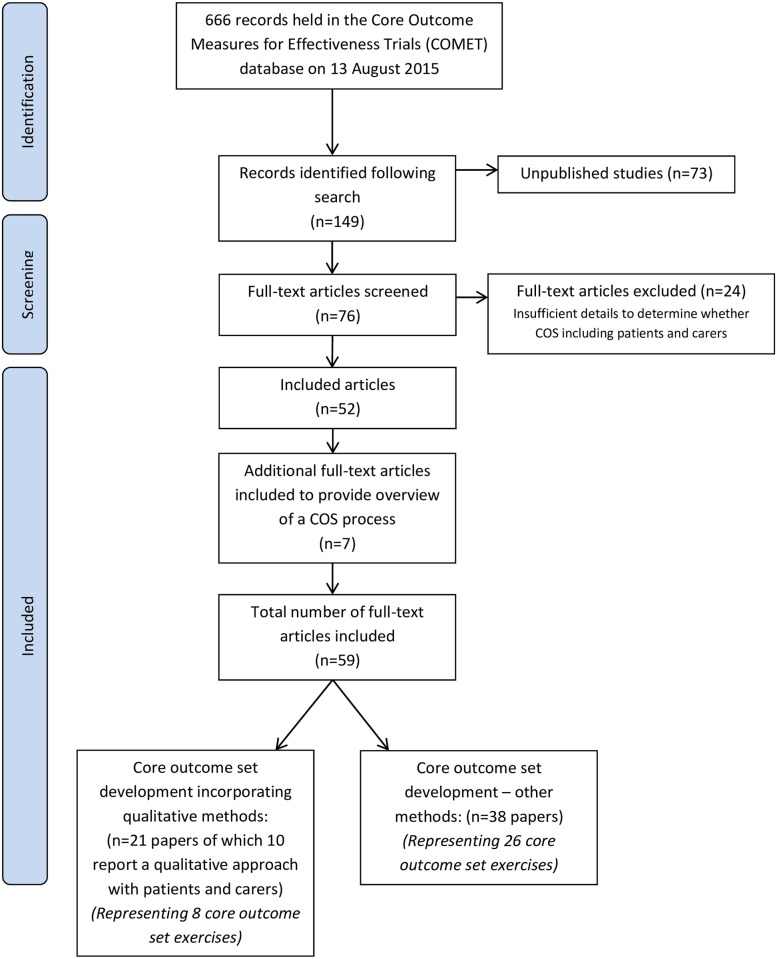
Flow diagram of paper identification and inclusion process.

Of the 666 records on the COMET database, our initial search strategy returned 149 papers (Fig 1). Seventy-three of the returned studies were unpublished, and of the remaining 76, 24 were excluded because there were insufficient details to determine whether they were reporting a COS including patients or carers. Through reference searches of the included papers an additional seven papers [16–22] were identified as part of the included COS development exercises. Of these, four [16, 19, 21, 22] were not archived on COMET at the point of the final search and three [17, 18, 20] papers were not returned in the initial search because they did not include patients or carers as participants; however, they described part of a COS pathway which included patient or carers reported in a separate publication.

#### Participants in the COS development

The 59 included papers represent a total of 34 COS development exercises covering 32 different health areas (Table 1). In 19 papers the number of participants was unclear.

**Table 1:** Summary of included papers.

Reference	Publication year	Health area	Ethics committee approval obtained	Data collection methods	Health Professionals (n)	Patients (n)	Carers and/or representatives (n)
**Core outcome set development exercises using qualitative methods with patients, carers and representatives**							
Allard et al [16]^α^	2014	Neurodisability	Not stated	Focus groups	0	50	47
				Interviews	0	4	6
Janssens [17] et al^α^	2014	Neurodisability	Not stated	Delphi	233	0	0
Morris et al [23] et al	2015	Neurodisability	Not stated	Other^Ω^	7	3	5
Arnold et al [24]	2008	Fibromyalgia	Yes	Focus groups	0	48	0
Mease et al [25]	2008	Fibromyalgia	Yes	Delphi	23	100	0
Mease et al [26]	2007	Fibromyalgia	Yes	Consensus meeting	Not stated	Not stated	0
Mease et al [27]	2009	Fibromyalgia	Not stated	Consensus conference	Not stated	Not stated	0
Stamm et al [21]^α^	2009	Osteoarthritis	Yes	Focus groups	0	56	0
Kloppenburg et al [28]	2014	Osteoarthritis	Not stated	Delphi	31	0	0
				Other^Ω^	Not stated	Not stated	0
*Potter et al* [19]^α^^π^	*2013*	*Breast cancer*	*Yes*	*Interviews*	*35*	*31*	*0*
*Potter et a* [20]^α^	*2014*	*Breast cancer*	*Yes*	*Interviews*	*35*	*0*	*0*
Potter et al [29]	2015	Breast cancer	Yes	Delphi	88	215	0
				Consensus meeting	23	15	0
Sanderson et al [30]	2010a	Rheumatoid arthritis	Yes	Interviews	0	23	0
Sanderson et al [31]	2010b	Rheumatoid arthritis	Yes	Nominal group technique	0	26	0
				Survey	0	254	0
*Swigris et al* [22]^α^	*2005*	*Interstitial lung disease (IPF)*	*Yes*	*Interviews*	*0*	*5*	*0*
				*Focus groups*	*0*	*15*	*0*
Saketkoo et al [32]	2014a	Interstitial lung disease (CTD)	Yes	Focus groups	0	45	0
				Delphi	254	0	0
Saketkoo et al [33]	2014b	Interstitial lung disease (CTD)	Yes	Nominal group technique	23	5	0
Tierney et al [34]	2013	Cleft palate, otitis media	Yes	Interviews	0	0	43
Tierney et al [35]^¥^	2015	Cleft palate, otitis media	Yes	Interviews	0	22	43^π^
Harman et al [36]	2015	Cleft palate, otitis media	Yes	Survey	0	8	35
				Delphi	104	0	0
				Consensus meeting	11	0	5
				Follow-up workshop	1	0	9
Turk et al [37]	2008	Chronic pain	Yes	Focus groups	0	31	0
				Survey	0	959	0
**Core outcome set development exercises using other methods**							
Bellm et al [38]	2002	Oral mucositis	Not stated	Other^Ω^	9	2	0
Bennett et al [39]	2012	Gestational diabetes mellitus	Not stated	Survey	4	0	2
				Consensus meeting	4	0	2
				Delphi	7	0	2
				Online evaluation	Not stated	0	Not stated
Broder et al [40]	2000	Uterine fibroids	Not stated	Delphi	9	0	1
				Nominal group technique	10	0	1
Buch et al [41]	2015	Rheumatic diseases	Not stated	Other^Ω^	20	2	0
				Delphi	20	1	0
Carlson et al [42]	2003	Mania/Bipolar disorder	Not stated	Consensus conference	53	0	Not stated
Chiarotto et al [43]	2015	Lower back pain	Exempt	Delphi	129	14	0
Chitnis et al [44]	2012	Multiple Sclerosis	Not stated	Survey	51	0	Not stated
Chitnis et al [45]	2013	Multiple Sclerosis	Not stated	Consensus meeting	69	0	Not stated
				Survey	Not stated	0	0
Devane et al [5]	2007	Maternity care	Yes	Delphi	194	9	15
Gladman et al [46]	2005	Psoriatic arthritis	Not stated	Nominal group technique	Not stated	Not stated	0
Gladman et al [47]	2007	Psoriatic arthritis	Not stated	Consensus meeting	Not stated*	Not stated*	0
Goldhahn et al [48]	2014	Distal radius fracture	Not stated	Nominal group technique	Not stated^0^	Not stated^0^	0
Gonzalez et al [49]	2011	Vitiligo	Not stated	Consensus meeting	Not stated	Not stated	0
Eleftheriadou et al [50]	2012	Vitiligo	Yes	Survey	Not stated	Not stated^Ψ^	0
Eleftheriadou et al [51]	2015	Vitiligo	Not stated	Delphi	69	32	0
Haeusler et al [52]	2015	Febrile neutropenia	Not stated	Delphi	39	4	0
Haywood et al [53]	2014	Hip fracture	Not stated	Survey	13	1	3
				Nominal group technique	22	0	3
Karas [54]	2015	Acute diarrhoea	Not stated	Delphi	70	0	31
Katona et al [55]	2007	Dementia	Not stated	Consensus conference	33	0	3
Merkies et al [56]	2006	Peripheral neuropathy	Not stated	Consensus conference	22	1	0
Moniz-Cook et al [57]	2008	Dementia care	Not stated	Consensus meeting	Not stated	0	0
				Survey	131	0	5
				Consensus meeting	Not stated	0	0
				Consensus meeting	Not stated	0	0
Reilly et al [58]	2006	Charcot-Marie-Tooth disease type 1A	Not stated	Consensus conference	21	2	0
Salaffi et al [59]	2012	Fibromyalgia	Yes	Delphi	252	86	0
Schmitt et al [60]	2007	Eczema	Not stated	Survey	6	4	2
Schmitt et al [61]	2010	Eczema	Not stated	Consensus conference	Not stated^#^	Not stated^#^	Not stated^#^
Schmitt et al [62]	2011	Eczema	Not stated	Delphi	40	6	0
Schmitt et al [63]	2012	Eczema	Not stated	Nominal group technique	38	5	0
Sinha et al [64]	2012	Asthma	Yes	Delphi	46	0	0
				Survey	0	11	88
Stuart et al [65]	2011	Ovarian cancer	Not stated	Consensus conference	Not stated	0	Not stated
Thigpen et al [66]	2011	Ovarian cancer	Not stated	Consensus conference	Not stated	0	Not stated
Tugwell et al [67]	1993	Rheumatoid arthritis	Not stated	Consensus conference	Not stated	Not stated	Not stated
				Nominal group technique	Not stated	Not stated	Not stated
Kirwan et al [68]	2003	Rheumatoid arthritis	Not stated	Consensus conference	46	11	0
Kirwan et al [69]	2005	Rheumatoid arthritis	Not stated	Consensus conference	160	19	0
Kirwan et al [70]	2007	Rheumatoid arthritis	Not stated	Consensus conference	60	20	0
Van der Heijde et al [71]	1997	Ankylosing spondylitis	Not stated	Other^Ω^	Not stated	Not stated	0
				Nominal group technique	Not stated	Not stated	0
Vargus-Adams et al [72]	2009	Cerebral palsy	Yes	Delphi	39	21	23
MacKichen et al [18]^α^	2015	Chronic pain after total knee replacement	Not stated	Focus groups	18	0	0
Wylde et el [73]	2015	Chronic pain after total knee replacement	Yes	Delphi	39	71	0
				Consensus meeting	Not stated	12	0

*total number of participants = 137, breakdown not provided.

^0^ total number of participants = 26, breakdown not provided.

^#^total number of participants = 40, breakdown not provided.

^Ψ^ total number of participants = 461, breakdown not provided.

^α^ Papers not in original search, included because they are part of the COS pathway.

^¥^ Full details given in Tierney 2013.

^Ω^ Other = meetings/semi-structured discussions.

Papers in italics have a qualitative component which was carried out prior to the COS exercise and was not specifically designed for the COS work but has fed into it.

Not Stated = participants in stakeholder group implied by numbers not given.

**N.B**. Papers grouped together are part of the same COS exercise.

The data collection methods used in COS development including patients and carers were; Delphi exercises (n = 14), consensus conferences/meetings (n = 20), surveys (n = 10), interviews (n = 6), focus groups (n = 6), nominal group techniques (n = 9) and other (n = 5). For the methods where participant numbers were reported fully, the mean percentage of patients and carers in each ranged from 20% to 86% (Table 2). Patients and carers formed the majority or all of the participants in surveys, interviews and focus groups. Health Professionals formed the majority of participants in nominal group techniques, Delphi exercises and consensus meetings. In all cases where qualitative data collection methods were utilised the primary aim was to identify outcomes of importance to the participants.

**Table 2:** Mean percentage of Health professionals to patients and carers by data collection method.

Method	Health Professionals (mean %)	Patients and carers (mean %)
Consensus meetings/conferences (n = 9)	80	20
Delphi (n = 19)	77	23
Nominal group technique (n = 5)	70	30
Surveys (n = 8)	36	64
Interviews (n = 7)	22	78
Focus groups (n = 7)	14	86

**N.B.** not all papers provided a full breakdown of participants and are therefore not included.

### Qualitative studies to inform COS development involving patients and carers

Ten papers [16, 19, 21, 22, 24, 30, 32, 34, 35, 37] reported using qualitative data collection methods to identify outcomes important to patients and carers (Table 3). However, of these only three were clearly reported as part of a COS development process [16, 24, 30]; five discussed outcomes with patients and carers but it was unclear whether the data were collected specifically with the intention to include them in a COS [21, 32, 34, 35, 37]; and two further studies were conducted for other primary research aims, such as exploring perceptions of access to care [19, 22].

**Table 3:** Reporting of qualitative methods with patients and carers.

	Allard 2014 [16]	Arnold 2008 [24]	Potter 2013 [19]	Saketkoo 2014a [32]	Swigris 2005 [22]	Stamm 2009 [21]	Sanderson 2010a [30]	Tierney 2013 [34]	Tierney 2015 [35]	Turk 2008 [37]
**Health area**	Neurodisability	Fibromyalgia	Breast cancer	Interstitial lung disease	Interstitial lung disease	Osteoarthritis	Rheumatoid arthritis	Cleft palate, otitis media	Cleft palate, otitis media	Chronic pain
**Theoretical framework**	N/R	N/R	N/R	N/R	N/R	N/R	N/R	N/R	N/R	N/R
**Sampling**										
*Approach*	Purposive	Purposive	Purposive	N/R^#^	Purposive	Purposive	Purposive	Purposive	Purposive	N/R^#^
*No*. *approached*	N/R	N/R	N/R	N/R	N/R	N/R	N/R	N/R	N/R	N/R
*No*. *taking part*	107	48	31	45	20	56	23	43	22	31
*Age*	Yes	Yes	Yes	Yes^Ψ^	Yes	Yes	Yes	Yes	Yes	Yes
*Gender*	Yes	Yes	Yes	Yes^Ψ^	Yes	Yes	Yes	Yes	Yes	Yes
*Ethnicity*	Yes	Yes	N/R	Yes^Ψ^	N/R	N/R	N/R	N/R	N/R	Yes
*Socio economic status*	Yes	N/R	N/R	N/R	N/R	N/R	N/R	N/R	N/R	Yes
*Clinically relevant info*	Yes	Yes	Yes	Yes^Ψ^	Yes	Yes	Yes	Yes	Yes	Yes
**Data collection**										
*Focus groups*	Yes	Yes	n/a	Yes	Yes	Yes	n/a	n/a	n/a	Yes
*Interviews*	Yes	n/a	Yes	n/a	Yes	n/a	Yes	Yes	Yes	n/a
**Data analysis**										
*A priori categories applied*	Yes (plus emergent themes)	No	No	No	No	No	No	No	No	No
Thematic/content analysis*	Yes	n/a	n/a	Yes	Yes	Yes	n/a	Yes	Yes	Yes
Grounded theory**	No	Yes	Yes	No	No	No	Yes	No	No	No
Framework approach***	Yes	No	No	No	No	No	Yes	Yes	Yes	No
*Data saturation mentioned*	Yes	No	Yes	No	Yes	No	Yes	No	No	No
**Triangulation**										
*Multiple coders/perspectives*	Yes	Yes	Yes	Yes	Yes	Yes	Yes	Yes	Yes	Yes
*Data collection methods (both focus groups and interviews)*	Yes	No	No	No	Yes	No	No	No	Yes	No
**Reflexivity**	No	Yes	Yes	No	No	No	No	No	No	No

N/R = not reported, n/a = not applicable,

^#^ = Some indication of types of participant included but not the sampling approach used.

^Ψ^ Details reported in Saketkoo 2014b.

* Paper describes a generic thematic / content approach

** Paper describes analysis informed by Grounded Theory approaches, rather than an explicit Grounded Theory methodological framework

*** Paper refers to the use of framework as part of the analytical process

#### Methodological framework

Of the papers reporting a qualitative approach in the development of a COS [16, 19, 21, 22, 24, 30, 32, 34, 35, 37] none explicitly reported an underpinning methodological framework.

#### Sampling

Eight papers [16, 19, 21, 22, 24, 30, 34, 35] reported using purposive sampling and two [32, 37] did not discuss a clear sampling strategy. All papers gave details of participant age, gender and some clinical detail (e.g. disease severity). However, some omitted details on socio economic status [19, 21, 22, 24, 30, 32, 34, 35] and ethnicity [19, 21, 22, 30, 34, 35]. Nine papers [16, 19, 22, 24, 30, 32, 34, 35, 37] recruited participants from a single country but one paper [21] included participants from five different European countries.

#### Data collection

Four papers reported interviews with patients and carers as the only data collection method [19, 30, 34, 35]. The number of interviews reported in these studies ranged from 22 to 31. Focus groups were the only method reported in four papers: Arnold [24] and Saketkoo [32] conducted six focus groups, Turk [37] four, and Stamm [21] ten. There was an average of seven participants per group. A combination of interviews and focus groups were reported in two papers; Allard [16] who carried out ten individual interviews and 12 focus groups (97 participants) and Swigris [22] who conducted five individual interviews and three focus groups (15 participants).

All studies were audio-recorded and transcribed verbatim, and all reported using topic or discussion guides. However, only four [16, 19, 30, 34] provided details of the contents or derivation of these.

#### Data analysis

Only one of the papers [16] reported the use of a priori categories in the analysis, with most using exclusively inductive data-driven approaches. Seven papers [16, 21, 22, 32, 34, 35, 37] reported using thematic or content analysis. Arnold [24], Potter [19] and Sanderson [30] reported analysis based upon the principles of grounded theory. Four papers also referred to the use of the framework approach in the analytical description [16, 30, 34, 35].

Four interview studies reported reaching data saturation [16, 19, 22, 30]. In two cases, saturation was judged in conjunction with focus group data [16, 22]. None of the papers reporting on focus groups only [21, 24, 32, 37] described achieving data saturation.

#### Triangulation

Data were analysed using multiple coders of and/or perspectives on the data in all of the included papers. Three [16, 22, 35] reported on the triangulation [74] of two different data collection methods (interviews and focus groups).

#### Reflexivity

Only two papers [19, 24] included any reflexive content. Potter [19] reported the use of a medically trained interviewer and reflected on the influence this may have had on the results. Arnold [24] discussed the use of an experienced facilitator with no prior knowledge of the condition under investigation, to avoid leading discussions.

#### Reported strengths and limitations

All studies acknowledged some strengths and limitations of their work. The main limitation noted in the included papers concerned recruitment. Five papers reported on the difficulty in recruiting an ethnically diverse and gender balanced sample [16, 24, 30, 33, 34]. Four discussed that they were unable to recruit participants to fulfil the desired sampling quota, for example, with not all categories of the disease/investigation under investigation being included [16, 24, 30, 37].

Sanderson [31] and Turk [37] reported that patients and carers identified a rather large and varied range of outcomes important to them, making subsequent rating and ranking very difficult. Potter [19] and Tierney [34] also highlighted the difficulty that participants may have when asked to recall their treatments and experience.

The participation of patients and carers in the core outcome set process was identified as a strength in 3 papers. Allard [16] stated that the differences between patients and parents were highlighted, and Sanderson [30] and Saketkoo [32] both reported that outcomes identified by patients and carers as important to them were not in current professional core outcome sets.

#### Resource use associated with qualitative methodology

Other than details on the length of the focus groups and interviews and reimbursements, very little information about the resources required to carry out qualitative data collection methods in COS development was reported in the included papers.

#### Planned and ongoing studies

As of 13 August 2015, 73 studies (S1 appendix) were registered on the COMET database as planned or ongoing COS development studies. Of these, all studies reported that they intended to include patients and carers as participants and 37 (52%) stated they wouldbe using qualitative methods as part of the COS pathway (S2 appendix). Of these 37, 21 planned to use both interviews and focus groups. Of the planned and ongoing studies five have published protocols [75–79]. Overall this supports the findings in Gorst’s [80] recently updated systematic review which reports the increase in COS development studies and the increasing involvement of patients and carers.

## Discussion

This study has described the different data collection methods used by COS developers in order to elicit patient and carer views on their preferred treatment outcomes, and has focused specifically on the reported use of methodology and methods within associated qualitative research. To our knowledge it is the first review to focus specifically on the use and reporting of qualitative research in COS development to date.

We have used the COMET database to identify COS studies. The database is based upon a systematic review of relevant studies and is regularly updated to ensure that new studies are added as they become available [4]. However, it is possible that there may be a lag time before studies are added to the database, or that COS relevant studies are not indexed or reported in ways that would mean they are captured within the database. We did perform reference searches of papers identified and found additional studies via this method. Therefore whilst the COMET database is an appropriate source of COS studies based on systematic review methodology, there is a possibility that there may be additional relevant studies not captured here. Furthermore, the focus of this review has been on participation in COS exercises. That is, we were concerned with the participation of patients and carers as research participants contributing data to the development of COSs. Of course patients and carers can also contribute to COS development via involvement in the research process as research partners and advisors, and in doing so influence the research and outputs. Our review has not focused on this involvement, which may not always be well reported and detailed in the outputs of COS studies. Further work to examine patient and carer involvement and its contribution to COS development would be useful. Recent work focused on patient and carer involvement demonstrates that this is a key issue that should be considered by researchers in the field [81].

To date, patients have participated in exercises that both identify relevant outcome domains for consideration in a COS, such as interviews, focus groups, and surveys, and also in the prioritisation and consensus methods that finalise a COS. However, the number and types of participants taking part in these, particularly patients and carers, were sometimes difficult to discern from the papers included in this review. Where participant numbers were reported, proportionately more patients participated in methods designed to identify relevant outcome domains for consideration, whilst Health Professionals were represented more than patients in prioritisation and consensus methods.

In this sample, qualitative and survey approaches have included patients more than Health Professionals to identify outcome domains. These methods may help incorporate patient perspectives that might otherwise go unheard, via the inclusion of patient preferred outcome domains in subsequent prioritisation and consensus methods. However, in prioritisation exercises (primarily Delphi methods in COS to date) where participant perspectives are aggregated and quantified then absolute numbers of participants from different stakeholder groups will influence outputs, particularly where views differ substantially between patients and carers and other stakeholder groups. Our observation that patients appear to be in the minority in these methods should be cause for reflection, although even if the number of participants from different stakeholder groups were balanced, there is some evidence to suggest that patients and carers rate many or all outcome domains as important in such exercises [31, 37]. If this were the case then other stakeholder views may dominate as the outcome domains they do not value, on aggregate, will not be taken forward to the final COS. The inclusion of qualitative research to incorporate patient and carer perspectives as a precursor to group consensus approaches will not necessarily guarantee that patients’ views are taken forward to the final COS.

In consensus meetings and conferences, one might expect Health Professionals to have more representation, as demonstrated here. The impact of patients and carers on outputs in these circumstances may well depend on the process and means by which their views are facilitated and accommodated, and on who takes part [81], as much as absolute numbers present versus other stakeholder groups.

Interestingly, one of the COS exercises we identified was expressly focused on understanding patient views and developing a patient core outcome set [30], building on the OMERACT work in rheumatoid arthritis. This approach does not rely on the integration of Health Professional and patient and carer views in a single COS exercise. Rather, it explicitly acknowledges that patient views are likely to be different to those of other stakeholders and therefore need specific consideration.

All of the qualitative research reviewed here was utilised as a means to ensure that patient and carer perspectives on outcome domains were accommodated in COS development processes. However, a key observation from this review is that some of this research (2 out of 10 papers) appear to have been designed and conducted for other primary research aims, not associated with COS development. Exceptions to this are Sanderson [30], Allard [16] and Arnold [24]. The remaining five papers [21, 32, 34, 35, 37] discussed outcomes with patients and carers but it is unclear whether the information was collected specifically with the intention to include it in a COS exercise. The availability of related research which is ready to feed into COS development, and the desire to include patient perspectives when specific COS-focused primary qualitative work is potentially time and resource intensive, could explain this. However, it does raise questions about the precise applicability of the underpinning qualitative research.

None of the papers stated a clear overarching qualitative methodological framework. Three [19, 24, 30] mentioned Grounded Theory, but only in descriptions of analytical approach. This may well be perfectly justifiable, for example if COS related qualitative research is being undertaken from an overtly generic qualitative research standpoint [82, 83], though to date there does not appear to have been any reflection on this in the primary COS papers, or the COS methodological literature. There are longstanding debates within the qualitative methodological literature [82, 83] about the use of methodological frameworks (e.g. grounded theory, phenomenology, ethnography), the coherency of underpinning research methods, and suitability for specific research aims. These have generally been in response to generic qualitative approaches that mix and match research methods, and which are more common in health-related research.

Reflection on methodology may help to further define the purpose and role of qualitative research in COS development. For example, if the primary aim is to understand and explain patients’ perspectives as a means to influence COS development then an approach such as Grounded Theory may be appropriate; if one argues that the lived experience of illness and treatment is of chief concern as it describes the essence of disease experience which we are trying to improve via trial research then a phenomenological framework may be informative; or if we are simply wanting a pragmatic precursor that lists ‘patient priorities’ from descriptive accounts prior to COS prioritisation exercises, then forms of generic descriptive qualitative research may suffice. Whilst to our knowledge these issues have not been considered in COS research, suitable methodological frameworks have been discussed in analogous outcome related work in the patient reported outcome development literature concerning content validity [84]. It would seem that there are very clear parallels between this work and COS qualitative research.

Thinking about data collection methods specifically, this review demonstrates the use of interviews, focus groups, and a combination of these. Whilst there has been some recent attention to this in the methodological literature [85] empirical comparisons of the outputs and relative merits of different methods is needed in this developing area. Some of this work is currently underway (S3 appendix).

The qualitative papers reviewed note limitations around recruitment and diversity of samples. The reporting of sample characteristics is also variable. This is important when considering the generalisability (transferability in qualitative terms) of findings. Implicitly, COS development for trials assumes the generalisability of the final COS to varied populations and settings [81]. Conversely, qualitative approaches may not necessarily lay claim to widespread generalisability, acknowledging that patients’ and carers’ perspectives may vary, for example, in time, place and according to cultural factors. In addition to clear reporting of sample characteristics to aid judgements of transferability this issue needs acknowledgment. This has been addressed in some COS work, for example with work to culturally validate a patient core set amongst non-white patients in rheumatoid arthritis [86]. Outcomes and their importance in health research is often a difficult concept for patients and carers to understand [16, 85]. The data collection methods used may have a direct impact on the depth of explanation of outcomes required [81]. For example, qualitative methods allow participants to talk about their experiences of illness without the need for an in-depth understanding of outcomes [87]. There is currently no guidance on how to discuss outcomes with patients and carers in qualitative research. The sharing of best practice and the publication of topic guides will aid future COS work [81, 85].

There are some fairly simple reporting recommendations (Table 4) that we would make for future qualitative COS work which include: clear reporting of aims in relation to the COS development; sampling and sample characteristics; data collection methods and derivation; use and reporting of the topic guide; data analysis; overt description of findings in the context of the COS; and reflection on strengths and limitations of approach. Beyond this we would suggest that there is a need for more fundamental consideration of the role of qualitative methods in COS and related methodological approaches, of the relative merits of different data collection approaches in terms of knowledge produced and time and resource requirements, as well as claims to generalisability.

**Table 4:** Reporting recommendations for qualitative research methods in COS development.

**1.**	Research aims and relationship with broader COS development process
**2.**	Sampling approach
**3.**	Type of data collection methods (e.g. interviews, focus groups, combination); content and derivation / justification (e.g. topic guide)
**4.**	Analytical approach and justification
**5.**	Sample characteristics and participants numbers
**6.**	Findings related to outcome domains (concordant with research aims)
**7.**	Report approaches to ensuring rigour (e.g. multiple perspectives on the data, respondent validation) and consider reflexive content
**8.**	Discuss strengths and limitations of approach

## Supporting information

S1 Appendix Planned and ongoing studies. (DOCX)

S2 Appendix Planned and ongoing studies using qualitative methods. (DOCX)

S3 Appendix Unpublished work. (DOCX)

S4 Appendix PRISMA statement. (DOC)

## References

[1] NHS Choices 2015 [updated 10 July 2015]. http://www.nhs.uk/Conditions/Clinical-trials/Pages/Introduction.aspx.

[2] NICE Nationl Institute for Health and Care Excellence [cited 2016 14 June]. https://www.nice.org.uk/.

[3] The National Institute for Health Research 2015 [2 April 2015]. http://www.nihr.ac.uk/about/mission-of-the-nihr.htm.

[4] COMET initiative 2011 [cited 2015 26 January]. http://www.comet-initiative.org/.

[5] Devane D, Begley CM, Clarke M, Horey D, Oboyle C. Evaluating Maternity Care: A Core Set of Outcome Measures. Birth. 2007;34(2):164–72. 10.1111/j.1523-536X.2006.00145.x 17542821

[6] Gargon E, Gurung B, Medley N, Altman DG, Blazeby JM, Clarke M, et al Choosing Important Health Outcomes for Comparative Effectiveness Research: A Systematic Review. PLoS One [Internet]. 2014; 9(6):[e99111 p.]. 10.1371/journal.pone.0099111 24932522 PMC4059640

[7] Clarke M. Standardising outcomes for clinical trials and systematic reviews. Trials. 2007;8(1):39. 18039365 10.1186/1745-6215-8-39 PMC2169261

[8] Whitehead L, Perkins G, Clarey A, Haywood K. A systematic review of the outcomes reported in cardiac arrest clinical trials: The need for a core outcome set. Resuscitation. 2015;Article in press. 10.1016/j.resuscitation.2014.11.013 25497393

[9] Clarke M, Williamson PR. Core outcome sets and systematic reviews. Sys Rev. 2016;5(1):1–4. 10.1186/s13643-016-0188-6 PMC4719739 26792080

[10] Williamson P, Altman D, Blazeby J, Clarke M, Devane D, Gargon E, et al Developing core outcome sets for clinical trials: issues to consider. Trials. 2012;13(1):132. 22867278 10.1186/1745-6215-13-132 PMC3472231

[11] Hewlett SA. Patients and clinicians have different perspectives on outcomes in arthritis. J Rheumatol. 2003;30(4):877–9. Epub 2003/04/03. 12672220

[12] Ritchie J, Lewis J, McNaughton Nicolls C, Ormston R, editors. Qualitative Research Practice: a Guide for Social Science Students and Researchers. 2nd ed ed. London: Sage; 2013.

[13] Gargon E, Williamson P, Altman DG, Blazeby J, Clarke M. The COMET Initiative database: progress and activities from 2011 to 2013. Trials. 2014;15:279 10.1186/1745-6215-15-279 25012001 PMC4107994

[14] Carter SM, Little M. Justifying Knowledge, Justifying Method, Taking Action: Epistemologies, Methodologies, and Methods in Qualitative Research Qual Health Res. 2007;17(10):1316–28. 10.1177/1049732307306927 18000071

[15] CASP Qualitative Checklist 2013 [5 October 2015]. http://www.casp-uk.net/#!casp-tools-checklists/c18f8.

[16] Allard A, Fellowes A, Shilling V, Janssens A, Beresford B, Morris C. Key health outcomes for children and young people with neurodisability: qualitative research with young people and parents. BMJ Open. 2014;4(4):e004611 Epub 2014/04/22. 10.1136/bmjopen-2013-004611 24747792 PMC3996811

[17] Janssens A, Williams J, Tomlinson R, Logan S, Morris C. Health outcomes for children with neurodisability: what do professionals regard as primary targets? Archives of disease in childhood. 2014;99(10):927–32. Epub 2014/05/24. 10.1136/archdischild-2013-305803 24854564

[18] MacKichan F, Wylde V, Gooberman-Hill R. Pathways Through Care for Long-Term Pain after Knee Replacement: A Qualitative Study of Healthcare Professionals. Musculoskeletal Care. 2015. 10.1002/msc.1093 25943433

[19] Potter S, Mills N, Cawthorn S, Wilson S, Blazeby J. Exploring inequalities in access to care and the provision of choice to women seeking breast reconstruction surgery: a qualitative study. British journal of cancer. 2013;109(5):1181–91. Epub 2013/08/10. 10.1038/bjc.2013.461 23928662 PMC3778305

[20] Potter S, Mills N, Cawthorn SJ, Donovan J, Blazeby JM. Time to be BRAVE: is educating surgeons the key to unlocking the potential of randomised clinical trials in surgery? A qualitative study. Trials. 2014;15:80 Epub 2014/03/19. 10.1186/1745-6215-15-80 24628821 PMC4003809

[21] Stamm T, van der Giesen F, Thorstensson C, Steen E, Birrell F, Bauernfeind B, et al Patient perspective of hand osteoarthritis in relation to concepts covered by instruments measuring functioning: a qualitative European multicentre study. Ann Rheum Dis. 2009;68(9):1453–60. Epub 2008/09/04. 10.1136/ard.2008.096776 18765429

[22] Swigris JJ, Stewart AL, Gould MK, Wilson SR. Patients' perspectives on how idiopathic pulmonary fibrosis affects the quality of their lives. Health and quality of life outcomes. 2005;3:61 Epub 2005/10/11. 10.1186/1477-7525-3-61 16212668 PMC1276807

[23] Morris C, Janssens A, Shilling V, Allard A, Fellowes A, Tomlinson R, et al Meaningful health outcomes for paediatric neurodisability: Stakeholder prioritisation and appropriateness of patient reported outcome measures. Health and quality of life outcomes. 2015;13:87 Epub 2015/06/26. 10.1186/s12955-015-0284-7 26108625 PMC4478638

[24] Arnold LM, Crofford LJ, Mease PJ, Burgess SM, Palmer SC, Abetz L, et al Patient perspectives on the impact of fibromyalgia. Patient Edu Couns. 2008;73(1):114–20. 10.1016/j.pec.2008.06.005. PMC2564867 18640807

[25] Mease PJ, Arnold LM, Crofford LJ, Williams DA, Russell IJ, Humphrey L, et al Identifying the clinical domains of fibromyalgia: Contributions from clinician and patient delphi exercises. Arthritis Care Res. 2008;59(7):952–60. 10.1002/art.23826 18576290

[26] Mease P, Arnold LM, Bennett R, Boonen A, Buskila D, Carville S, et al Fibromyalgia syndrome. J Rheumatol. 2007;34(6):1415–25. 17552068

[27] Mease P, Arnold LM, Choy EH, Clauw DJ, Crofford LJ, Glass JM, et al Fibromyalgia Syndrome Module at OMERACT 9: Domain Construct. J Rheumatol. 2009;36(10):2318–29. 10.3899/jrheum.090367 19820221 PMC3419373

[28] Kloppenburg M, Boyesen P, Smeets W, Haugen IK, Liu R, Visser W, et al Report from the OMERACT Hand Osteoarthritis Special Interest Group: advances and future research priorities. J Rheumatol. 2014;41(4):810–8. Epub 2014/01/17. 10.3899/jrheum.131253 24429165

[29] Potter S, Holcombe C, Ward JA, Blazeby JM. Development of a core outcome set for research and audit studies in reconstructive breast surgery. The British journal of surgery. 2015. Epub 2015/07/17. 10.1002/bjs.9883 PMC5034747 26179938

[30] Sanderson T, Morris M, Calnan M, Richards P, Hewlett S. What outcomes from pharmacologic treatments are important to people with rheumatoid arthritis? Creating the basis of a patient core set. Arthritis Care Res. 2010a;62(5):640–6. 10.1002/acr.20034 PMC2887082 20461785

[31] Sanderson T, Morris M, Calnan M, Richards P, Hewlett S. Patient perspective of measuring treatment efficacy: The rheumatoid arthritis patient priorities for pharmacologic interventions outcomes. Arthritis Care Res. 2010b;62(5):647–56. 10.1002/acr.20151 PMC2886964 20461786

[32] Saketkoo LA, Mittoo S, Frankel S, LeSage D, Sarver C, Phillips K, et al Reconciling healthcare professional and patient perspectives in the development of disease activity and response criteria in connective tissue disease-related interstitial lung diseases. J Rheumatol. 2014a;41(4):792–8. Epub 2014/02/04. 24488412 10.3899/jrheum.131251 PMC4369780

[33] Saketkoo LA, Mittoo S, Huscher D, Khanna D, Dellaripa PF, Distler O, et al Connective tissue disease related interstitial lung diseases and idiopathic pulmonary fibrosis: provisional core sets of domains and instruments for use in clinical trials. Thorax. 2014b;69(5):428–36. Epub 2013/12/26. 24368713 10.1136/thoraxjnl-2013-204202 PMC3995282

[34] Tierney S, O'Brien K, Harman NL, Madden C, Sharma RK, Callery P. Risks and benefits of ventilation tubes and hearing aids from the perspective of parents of children with cleft palate. International journal of pediatric otorhinolaryngology. 2013;77(10):1742–8. Epub 2013/09/07. 10.1016/j.ijporl.2013.08.006 24007893

[35] Tierney SP, O'Brien KP, Harman NLP, Sharma RKF, Madden CMD, Callery PP. Otitis Media With Effusion: Experiences of Children With Cleft Palate and Their Parents. Cleft Palate Craniofac J. 2015;52(1):23–30. 10.1597/13-139 24237229

[36] Harman NL, Bruce IA, Kirkham JJ, Tierney S, Callery P, O'Brien K, et al The Importance of Integration of Stakeholder Views in Core Outcome Set Development: Otitis Media with Effusion in Children with Cleft Palate. PLoS One. 2015;10(6):e0129514 Epub 2015/06/27. 10.1371/journal.pone.0129514 26115172 PMC4483230

[37] Turk DC, Dworkin RH, Revicki D, Harding G, Burke LB, Cella D, et al Identifying important outcome domains for chronic pain clinical trials: An IMMPACT survey of people with pain. Pain. 2008;137(2):276–85. 10.1016/j.pain.2007.09.002. 17937976

[38] Bellm LA, Cunningham G, Durnell L, Eilers J, Epstein JB, Fleming T, et al Defining Clinically Meaningful Outcomes in the Evaluation of New Treatments for Oral Mucositis: Oral Mucositis Patient Provider Advisory Board. Cancer Invest. 2002;20(5–6):793–800. 12197238 10.1081/cnv-120002497

[39] Bennett WL, Robinson KA, Saldanha IJ, Wilson LM, Nicholson WK. High Priority Research Needs for Gestational Diabetes Mellitus. J Womens Health. 2012;21(9):925–32. 10.1089/jwh.2011.3270 PMC3654820 22747422

[40] Broder MS, Landow WJ, Goodwin SC, Brook RH, Sherbourne CD, Harris K. An Agenda for Research into Uterine Artery Embolization: Results of an Expert Panel Conference. J Vasc Interv Radiol. 2000;11(4):509–15. 10.1016/S1051-0443(07)61386-4. 10787212

[41] Buch MH, Silva-Fernandez L, Carmona L, Aletaha D, Christensen R, Combe B, et al Development of EULAR recommendations for the reporting of clinical trial extension studies in rheumatology. Ann Rheum Dis. 2015;74(6):963–9. Epub 2014/05/16. 10.1136/annrheumdis-2013-204948 24827533 PMC4431343

[42] Carlson GA, Jensen PS, Findling RL, Meyer RE, Calabrese J, DelBello MP, et al Methodological Issues and Controversies in Clinical Trials with Child and Adolescent Patients with Bipolar Disorder: Report of a Consensus Conference. J Child Adolesc Psychopharmacol. 2003;13(1):13–27. 10.1089/104454603321666162. 12804123

[43] Chiarotto A, Deyo RA, Terwee CB, Boers M, Buchbinder R, Corbin TP, et al Core outcome domains for clinical trials in non-specific low back pain. Eur Spine J. 2015;24(6):1127–42. Epub 2015/04/07. 10.1007/s00586-015-3892-3 25841358

[44] Chitnis T, Tenembaum S, Banwell B, Krupp L, Pohl D, Rostasy K, et al Consensus statement: evaluation of new and existing therapeutics for pediatric multiple sclerosis. Mult Scler. 2012;18(1):116–27. 10.1177/1352458511430704 22146610

[45] Chitnis T, Tardieu M, Amato MP, Banwell B, Bar-Or A, Ghezzi A, et al International Pediatric MS Study Group Clinical Trials Summit: Meeting report. Neurology. 2013;80(12):1161–8. 10.1212/WNL.0b013e318288694e 23509048 PMC3662305

[46] Gladman DD. Consensus exercise on domains in psoriatic arthritis. Ann Rheum Dis. 2005;64(suppl 2):ii113–ii4. 15708924 10.1136/ard.2004.030882 PMC1766862

[47] Gladman DD, Mease PJ, Strand V, Healy P, Helliwell PS, Fitzgerald O, et al Consensus on a core set of domains for psoriatic arthritis. J Rheumatol. 2007;34(5):1167–70. 17477480

[48] Goldhahn J, Beaton D, Ladd A, Macdermid J, Hoang-Kim A. Recommendation for measuring clinical outcome in distal radius fractures: a core set of domains for standardized reporting in clinical practice and research. Archives of orthopaedic and trauma surgery. 2014;134(2):197–205. Epub 2013/06/04. 10.1007/s00402-013-1767-9 23728832

[49] Gonz ález U, Whitton M, Eleftheriadou V, Pinart M, Batchelor J, Leonardi-Bee J. Guidelines for designing and reporting clinical trials in vitiligo. Arch Dermatol. 2011;147(12):1428–36. 10.1001/archdermatol.2011.235 21844427

[50] Eleftheriadou V, Thomas KS, Whitton ME, Batchelor JM, Ravenscroft JC. Which outcomes should we measure in vitiligo? Results of a systematic review and a survey among patients and clinicians on outcomes in vitiligo trials. The British journal of dermatology. 2012;167(4):804–14. 10.1111/j.1365-2133.2012.11056.x 22591025

[51] Eleftheriadou V, Thomas K, van Geel N, Hamzavi I, Lim H, Suzuki T, et al Developing core outcome set for vitiligo clinical trials: international e-Delphi consensus. Pigment Cell Melanoma Res. 2015;28(3):363–9. Epub 2015/02/04. 10.1111/pcmr.12354 25645179

[52] Haeusler GM, Phillips RS, Lehrnbecher T, Thursky KA, Sung L, Ammann RA. Core outcomes and definitions for pediatric fever and neutropenia research: a consensus statement from an international panel. Pediatric blood & cancer. 2015;62(3):483–9. Epub 2014/12/03. 25446628 10.1002/pbc.25335

[53] Haywood KL, Griffin XL, Achten J, Costa ML. Developing a core outcome set for hip fracture trials. Bone Joint J. 2014;96-B(8):1016–23. 10.1302/0301-620X.96B8.33766 25086115

[54] Karas J, Ashkenazi S, Guarino A, Lo Vecchio A, Shamir R, Vandenplas Y, et al A core outcome set for clinical trials in acute diarrhoea. Archives of disease in childhood. 2015;100(4):359–63. Epub 2014/11/22. 10.1136/archdischild-2014-307403 25414251

[55] Katona C, Livingston G, Cooper C, Ames D, Brodaty H, Chiu E. International Psychogeriatric Association consensus statement on defining and measuring treatment benefits in dementia. Int Psychogeriatr. 2007;19(03):345–54. 17386120 10.1017/S1041610207005145

[56] Merkies ISJ, Lauria G. 131st ENMC International workshop: Selection of Outcome Measures for Peripheral Neuropathy Clinical Trials: 10–12 December 2004, Naarden, The Netherlands. Neuromuscul Dis. 2006;16(2):149–56. 10.1016/j.nmd.2005.12.003. 16431105

[57] Moniz-Cook E, Vernooij-Dassen M, Woods R, Verhey F, Chattat R, Vugt MD, et al A European consensus on outcome measures for psychosocial intervention research in dementia care. Aging Ment Health. 2008;12(1):14–29. 10.1080/13607860801919850 18297476

[58] Reilly MM, de Jonghe P, Pareyson D. 136th ENMC International Workshop: Charcot–Marie–Tooth Disease Type 1A (CMT1A)8–10 April 2005, Naarden, The Netherlands. Neuromuscular disorders: NMD. 2006;16(6):396–402. 10.1016/j.nmd.2006.03.008. 16684603

[59] Salaffi F, Ciapetti A, Sarzi Puttini P, Atzeni F, Iannuccelli C, Di Franco M, et al Preliminary identification of key clinical domains for outcome evaluation in fibromyalgia using the Delphi method: the Italian experience. Reumatismo. 2012;64(1). 10.4081/reumatismo.2012.27 22472780

[60] Schmitt J, Langan S, Williams HC. What are the best outcome measurements for atopic eczema? A systematic review. J Allergy and Clin Immunol. 2007;120(6):1389–98. 10.1016/j.jaci.2007.08.011. 17910890

[61] Schmitt J, Williams H. Harmonising Outcome Measures for Eczema (HOME). Report from the First International Consensus Meeting (HOME 1), July 2010, Munich, Germany. The British journal of dermatology. 2010;163(6):1166–8. 21137114 10.1111/j.1365-2133.2010.10054.x

[62] Schmitt J, Langan S, Stamm T, Williams HC. Core Outcome Domains for Controlled Trials and Clinical Recordkeeping in Eczema: International Multiperspective Delphi Consensus Process. J Invest Dermatol. 2011;131(3):623–30. http://www.nature.com/jid/journal/v131/n3/suppinfo/jid2010303s1.html. 10.1038/jid.2010.303 20944653

[63] Schmitt J, Spuls P, Boers M, Thomas K, Chalmers J, Roekevisch E, et al Towards global consensus on outcome measures for atopic eczema research: results of the HOME II meeting. Allergy. 2012;67(9):1111–7. 10.1111/j.1398-9995.2012.02874.x 22844983

[64] Sinha IP, Gallagher R, Williamson P, Smyth R. Development of a core outcome set for clinical trials in childhood asthma: a survey of clinicians, parents, and young people. Trials. 2012;13(1):103. 22747787 10.1186/1745-6215-13-103 PMC3433381

[65] Stuart GCE, Kitchener H, Bacon M, duBois A, Friedlander M, Lederman J, et al 2010 Gynecologic Cancer InterGroup (GCIG) Consensus Statement on Clinical Trials in Ovarian Cancer. Int J Gynecol Cancer. 2011;21(4):750–5. 10.1097/IGC.0b013e31821b2568 21543936

[66] Thigpen T, DuBois A, McAlpine J, DiSaia P, Fujiwara K, Hoskins W, et al First-Line Therapy in Ovarian Cancer Trials. Int J Gynecol Cancer. 2011;21:756–62. 10.1097/IGC.0b013e31821ce75d 21543937

[67] Tugwell P, Boers M. Developing consensus on preliminary core efficacy endpoints for rheumatoid arthritis clinical trials. OMERACT committee. J Rheumatol. 1993;20(3):555–6. 8478872

[68] Kirwan J, Heiberg T, Hewlett S, Hughes R, Kvien T, Ahlmèn M, et al Outcomes from the Patient Perspective Workshop at OMERACT 6. J Rheumatol. 2003;30(4):868–72. 12672218

[69] Kirwan JR, Hewlett SE, Heiberg T, Hughes RA, Carr M, Hehir M, et al Incorporating the patient perspective into outcome assessment in rheumatoid arthritis—progress at OMERACT 7. J Rheumatol. 2005;32(11):2250–6. 16265712

[70] Kirwan JR, Minnock P, Adebajo A, Bresnihan B, Choy E, de Wit M, et al Patient perspective: fatigue as a recommended patient centered outcome measure in rheumatoid arthritis. J Rheumatol. 2007;34(5):1174–7. 17477482

[71] van der Heijde D, Bellamy N, Calin A, Dougados M, Khan MA, van der Linden S. Preliminary core sets for endpoints in ankylosing spondylitis. Assessments in Ankylosing Spondylitis Working Group. J Rheumatol. 1997;24(11):2225–9. 9375888

[72] Vargus-Adams JN, Martin LK. Measuring What Matters in Cerebral Palsy: A Breadth of Important Domains and Outcome Measures. Arch Phys Med Rehabil. 2009;90(12):2089–95. 10.1016/j.apmr.2009.06.018. 19969173

[73] Wylde V, MacKichan F, Bruce J, Gooberman-Hill R. Assessment of chronic post-surgical pain after knee replacement: development of a core outcome set. European journal of pain (London, England). 2015;19(5):611–20. Epub 2014/08/27. 10.1002/ejp.582 PMC4409075 25154614

[74] Carter N, Bryant-Lukosius D, DiCenso A, Blythe J, Neville AJ. The use of triangulation in qualitative research. Oncol Nurs Forum. 2014;41(5):545–7. Epub 2014/08/28. 10.1188/14.ONF.545-547 25158659

[75] Duffy JMN, van ‘t Hooft J, Gale C, Brown M, Grobman W, Fitzpatrick R, et al A protocol for developing, disseminating, and implementing a core outcome set for pre-eclampsia. Pregnancy Hypertens. 10.1016/j.preghy.2016.04.008. 27939467

[76] Kaiser U, Kopkow C, Deckert S, Sabatowski R, Schmitt J. Validation and application of a core set of patient-relevant outcome domains to assess the effectiveness of multimodal pain therapy (VAPAIN): a study protocol. BMJ Open. 2015;5(11):e008146 Epub 2015/11/08. 10.1136/bmjopen-2015-008146 26547084 PMC4636634

[77] Keeley T, Khan H, Pinfold V, Williamson P, Mathers J, Davies L, et al Core outcome sets for use in effectiveness trials involving people with bipolar and schizophrenia in a community-based setting (PARTNERS2): study protocol for the development of two core outcome sets. Trials. 2015;16(1):47. Epub 2015/04/19. 25887033 10.1186/s13063-015-0553-0 PMC4334396

[78] MacLennan S, Bekema HJ, Williamson PR, Campbell MK, Stewart F, MacLennan SJ, et al A core outcome set for localised prostate cancer effectiveness trials: protocol for a systematic review of the literature and stakeholder involvement through interviews and a Delphi survey. Trials. 2015;16(1):76. Epub 2015/04/19. 25887437 10.1186/s13063-015-0598-0 PMC4355995

[79] Waters AM, Tudur Smith C, Young B, Jones TM. The CONSENSUS study: protocol for a mixed methods study to establish which outcomes should be included in a core outcome set for oropharyngeal cancer. Trials. 2014;15:168 Epub 2014/06/03. 10.1186/1745-6215-15-168 24885068 PMC4023496

[80] Gorst SL, Gargon E, Clarke M, Blazeby JM, Altman DG, Williamson PR. Choosing Important Health Outcomes for Comparative Effectiveness Research: An Updated Review and User Survey. PLoS ONE. 2016;11(1):e0146444 10.1371/journal.pone.0146444 26785121 PMC4718543

[81] Young B, Bagley H. Including patients in core outcome set development: issues to consider based on three workshops with around 100 international delegates. Research Involvement and Engagement. 2016;2(1):1–13. 29507761 10.1186/s40900-016-0039-6 PMC5831887

[82] Caelli K, Ray L, Mill J. 'Clear as Mud': Toward greater clarity in generic qualitative research. Int J Qual Methods. 2003;2(2):1–24.

[83] Kahlke R. Generic qualitative approaches: pitfalls and benefits of methodological mixology. Int J Qual Methods 2014;13:37–52.

[84] Lasch KE, Marquis P, Vigneux M, Abetz L, Arnould B, Bayliss M, et al PRO development: rigorous qualitative research as the crucial foundation. Qual Life Res. 2010;19(8):1087–96. Epub 2010/06/01. 10.1007/s11136-010-9677-6 20512662 PMC2940042

[85] Keeley T, Williamson P, Callery P, Jones LL, Mathers J, Jones J, et al The use of qualitative methods to inform Delphi surveys in core outcome set development. Trials. 2016;17(1):1–9. 27142835 10.1186/s13063-016-1356-7 PMC4855446

[86] Sanderson T, Hewlett S, Calnan M, Morris M, Raza K, Kumar K. Exploring the cultural validity of rheumatology outcomes. Br J Nurs. 2012;21(17):1015–20, 522–3. Epub 2012/11/06. 10.12968/bjon.2012.21.17.1015 23123747

[87] Mathers J, Keeley T, Jones L, Calvert M, Williamson P, Jones J, et al Using qualitative research to understand what outcomes matter to patients: direct and indirect approaches to outcome elicitation. Trials. 2015;16(2):1-.25971836

