# Supplementary material for: A review of patient and carer participation and the use of qualitative research in the development of core outcome sets
Source: PLoS One. 2017 Mar 16;12(3):e0172937. doi: 10.1371/journal.pone.0172937 (PMC5354261; doi:10.1371/journal.pone.0172937)
Supplement: S1 Appendix — (DOCX) [file pone.0172937.s001.docx]

**S1 Appendix** - **Planned and ongoing studies**

1. Alam M, Maher I, Sobanko J, Cartee T. Core Outcome Set for the Appearance of Facial Aging.
2. Azuara-Blanco A, Hogg R, Boylan NLJ. Development of core outcomes measures for diabetic retinopathy interventions.
3. Azuara-Blanco A, Hogg R, Chakravarthy U. Development of core outcome measures for age-related macular degeneration (AMD) interventions.
4. Azuara-Blanco A, Ramsay C, R I. Development of core outcome measures for glaucoma interventions.
5. Beeckman D, Kottner Charite J. Developing a Core Outcome Set for Incontinence-Associated Dermatitis (IAD) Research.
6. Benstoem C, Moza A, Stoppe C, Goetzenich A, Autschbach R. Development of a core outcome set (COS) for non-minimal-invasive on-pump cardiothoracic clinical trials.
7. Birchwood M, Calvert M, Keeley T, Pinfold V, Davies L, England E, et al. PARTNERS 2: Development of a core outcome set for use in mental health trials involving people with schizophrenia or bipolar disorder in a community based setting.
8. Blazeby J, Brookes ST, Avery K, McNair AGK, Whistance RN. Development of core outcome sets for informed consent and clinical trials of colorectal surgery.
9. Byng R, Shaw J, Stirzaker A, Stewart AK, Quinn C, Lennox C, et al. Engager 2: Developing and evaluating a collaborative care intervention for offenders with common mental health problems, near to and after release.
10. Cartee T, Maher I, Sobanko J, Alam M. Core Outcome Set for Facial Structure and Function Post-Skin Cancer Excision.
11. Chazapis M, Moonesinghe R, Kamming D. Core Outcomes in Regional Anaesthesia (CORE).
12. Clark M, Samuelson E, Schaeffer M, Alam M. Core Outcome Set for Acne Scarring.
13. Colavincenzo M, Schlessinger D, Iyengar S, Samuelson E, Schaeffer M, Alam M. Core Outcome Set for Hair Loss/Non-Scarring Alopecia.
14. Costa M, Fernandez M, Tutton L, Khan U, Jain A, Kelly M, et al. A core outcome set for comparative effectiveness research in open lower limb fractures.
15. Coulman K, Owen-Smith A, Blazeby J, Welbourn R, Andrews R. the patient perspective of living with surgery for morbid obesity: Creating a patient "core" outcome set, and investigating ways to improve follow-up care.
16. Davidson B, Gurusamy K, Simillis C. Development of core outcome sets in clinical trials comparing treatments for the management of colorectal liver metastases and liver resection.
17. Denniston A, Calvert M, Murray P, Moore D, Mathers J, Blazeby J, et al. Defining a Core Outcome Set for Efficacy Trials in Adult Patients with Posterior Segment-Involving Uveitis.
18. Devane D, Smith V, Boylan G, Alfirevic Z, Kenny L. Developing a core outcome set (COS) for intrapartum fetal assessment.
19. Devane D, Smith V, Daly D, Lundgren I, Eri T, Begley CM, et al. Salutogenic Intrapartum Core Outcomes (SIPCO): Identification of a minimum dataset using an international eDelphi consensus process.
20. Devane D, Smith V, Dunne F. Developing a core outcome set (COS) for clinical trials in GDM.
21. Devane D, Smith V, Kenny L. Developing a core outcome set (COS) for clinical trials in intrauterine growth restriction.
22. Duffy J, Hirsch M, Davis C, Farquhar C. Developing a Core Outcome Set in Endometriosis.
23. Duffy JMN, McManus R, Ziebland S, Khan K, Altman DG, Fitzpatrick R, et al. Developing a core outcome set for hypertensive disorders in pregnancy.
24. Duncan-Millar J, Ali M, Pollock A, van Wijck F. Standardisation of Outcome Measures in Trials of Upper-Limb Rehabilitation after Stroke.
25. Egan A, Smith V, Devane D, Dunne F. Developing a core outcome set (COS) for clinical studies of prepregnancy care for women with pregestational diabetes mellitus.
26. Fabricius M, Heer R, Pickard R, McColl E. Development of core outcome measures for clinical trials in advanced prostate cancer.
27. Fernandes RM, Offringa M, Van der Lee JH, Klassen T, Hartling L, Plint A. Outcomes in clinical trials of bronchiolitis.
28. Forster A, Young J, Brown L, Crocker T, Clarke D, Clegg A. Core outcome set relevant to (physical rehabilitation with) frail older people (in care homes).
29. Gagnier J, Morgenstern H, MacEachern M. Core outcome measures for rotator cuff disorders.
30. Glenny A, Worthington H, Brocklehurst P, Taylor J, Riley P, Walsh T. Core outcome measures and selective outcome reporting in randomised controlled trials of oral medicine.
31. Glenny A, Worthington H, Walsh T, Burnside G. Core outcome measures and selective outcome reporting in randomised controlled trials for the prevention and treatment of periodontal disease.
32. Goncalves ACV, Samuel D, Demain S, Marques A, Cruz J. Development if a core outcome set to evaluate physical activity in people with dementia.
33. Hall D. Developing a global consensus on outcome measures for clinical trials in tinnitus: the COMiT initiative (Core Outcome Measures in Tinnitus).
34. Hall N, Ross A. Establishing a core outcome set (COS) for infants born with gastroschisis.
35. Harrop E. Supporting people bereaved through advanced illness: A systematic review of the evidence and development of a core outcome set for bereavement research in palliative care.
36. Hart N, Connolly B, McAuley D, Gulliford M. Development of a core outcome set for trials of rehabilitation following critical illness.
37. Iyengar S, Samuelson E, Schaeffer M, Alam M. Core Outcome Set for Rosacea.
38. Kaiser U, Schmitt J, Kopkow C, Sabatowski R. Validation and application of a core set of patient-relevant outcome domains to assess the effectiveness of multimodal pain therapy.
39. Kapadia M, Offringa M, Balasingham C, Joachim K, Cohen E, Mahant S, et al. Core set of outcomes for children with neuro-disability and gastrostomy tube dependency: A tool of standardized outcomes for clinical research and practice.
40. Kaufman J, Hill S, Ryan R, Lewin S, Bosch-Capblanch X, Cliff J, et al. Developing an outcomes taxonomy and preliminary core outcomes set for evaluating communication-related interventions for childhood vaccination (the COMMVAC2 study).
41. Khan K, Alfirevic Z, Saade GR, Mol BW, van't Hooft J. Defining core outcomes for studies on prevention of preterm birth in high risk women: COPOP project.
42. Knight S, Friend P, Blazeby J, McNair AGK, Avery K, Caskey F. Developing core outcome sets for trials of immunosuppression in kidney transplant recipients.
43. Lam T, N'Dow J, MacLennan S, Ramsay C, Campbell M, Entwistle V. Development of core outcomes for surgical management of localised prostate cancer to support decision-making by patients, clinicians and policy makers (Prostate Cancer Core Outcomes Project).
44. Marson T, Jacoby A, Hughes D, Kierans C, Cooper M, Williamson P, et al. Defining patient preferences and priorities for treatment options and outcomes in epilepsy.
45. McConachie H, Williams K, Le Couteur A, Charman T. MeASURe: Measurement in autism spectrum disorder under review.
46. Meher S, Alfirevic Z, Williamson P, Kirkham JJ. Core Outcome Sets for Prevention and Treatment of Postpartum Haemorrhage.
47. Myles P, Moonesinghe R, Boney O. Core Outcome Measures in Perioperative and Anaesthetic Care (COMPAC).
48. Nixon J. Outcomes for Pressure Ulcer Trials (OUTPUT).
49. Perez R, Moseley L, Harden N, Brunner F. A multi-centre international collaboration for the development and implementation of a core data set of outcome measures for Complex Regional Pain Syndrome clinical trials.
50. Rees J, Blazeby J. Development of core outcome sets (COS) for liver surgery for metastatic disease to use in clinical trials and national audit. - LIVCoS.
51. Rees JRE, Coolsen MME, Brookes ST, Avery K, Finch-Jones MD, Jong C, et al. Development of a core outcome set to assess the benefits and adverse outcomes after pancreatic surgery in clinical trials.
52. Reilly S, Wang Y, Morbey H, Leroi I, Williamson P, Keady J, et al. The development of a dementia core outcome set for dementia care in the community
53. Riley P, Glenny A, Worthington H, Walsh T. Core outcome measures and selective outcome reporting in randomised controlled trials (RCTs) of the management of oral mucositis in cancer patients.
54. Ringrow S, Blackwood B, McAuley D, Clarke M, Rose L. Standardizing reporting of core outcome measures in ventilation studies.
55. Schwendicke F, Innes N, Lamont T. Outcomes in Trials for Management of Caries Lesions (OuTMaC).
56. Semple MG, Sinha IP. Identifying common outcome measures for epidemic and pandemic studies of severe acute respiratory infection.
57. Shokeen D, Samuelson E, Schaeffer M, Alam M. Core Outcome Set for Post-Inflammatory Hyperpigmentation (PIH).
58. Smith P, Coomarasamy A, Clark J, Ismail K, Khan K. Core outcome measures for surgical management of miscarriage.
59. Sousa Dos Santos F, Thangaratinam S, Hogg M. Development of a Core Outcome Set for trials on Induction of Labour.
60. Sun Y, Yu C, He L, Fan J. Core Outcome Sets of integrity of modern and Traditional Chinese Medicine on treatment of chronic Hepatitis B.
61. T J, Tudur Smith C, Waters A. Head and neck cancer: defining core outcome sets for clinical trials
62. Tang J, Samuelson E, Schaeffer M, Alam M. Core Outcome Set for Melasma.
63. Thangaratinam S, Khan K, Pirie A, Al Watter BH, McCorrey D, Bagary M, et al. Defining core outcomes for clinical trials in pregnant women with epilepsy (E-core): A Delphi survey.
64. Thiboutot D, Tan J, Layton A. Development of Clinical Trials Outcome Instruments for Acne Vulgaris.
65. Vasic J, Samuelson E, Goldberg L, Alam M. Core Outcome Set for Actinic Keratosis.
66. Vasic J, Samuelson E, Schaeffer M, Alam M. Core Outcome Set for Treatment of Leg Veins.
67. Wallace S, Worrall L, Rose T, Le Dorze G. Improving Research Outcome Measurement in Aphasia (ROMA): Development of a Core Outcome Set.
68. Whitehead L, Perkins G, Haywood K, Finn J, Jacobs I, Brett S, et al. the development of a core outcome set for cardiac arrest clinical trials.
69. Zajicek J, Hobart J, Wright D, Wilcock G, Ritchie C, Counsell C, et al. Clinical Trials Methods in Neurodegenerative Diseases.
70. Zha A, Samuelson E, Schaeffer M, Alam M. Core Outcome Set for Scar.
71. Zhang J, Xing D, Shang H. Developing a Core Outcome Set for Traditional Chinese Medicine for Stable Angina Pectoris.
72. Zhang Y, Du L, Fan H. Core outcome sets for tuberculosis.
73. Zoet DA, van Rijn BB, Koster MPH, Franx A, Maas AHEM, Groot CJM. Primary endpoints in cohort studies evaluating cardiovascular disease risk after reproductive disorders.
