# Supplementary material for: A review of patient and carer participation and the use of qualitative research in the development of core outcome sets
Source: PLoS One. 2017 Mar 16;12(3):e0172937. doi: 10.1371/journal.pone.0172937 (PMC5354261; doi:10.1371/journal.pone.0172937)
Supplement: S2 Appendix — (DOCX) [file pone.0172937.s002.docx]

**S2 Appendix - Planned and ongoing studies using qualitative methods**

| **Author** | **Health area** | **Proposed sampling** | **Proposed data collection methods** | **Proposed analytical approach** |
| --- | --- | --- | --- | --- |
| Duffy (protocol available) | Pre-eclampsia | Not stated | Systematic review | Thematic content analysis |
|  |  |  | Interviews |  |
|  |  |  | Delphi |  |
| Kaiser (protocol available) | Chronic pain | Not stated | Delphi process | Not stated |
|  |  |  | Interview |  |
|  |  |  | Systematic review |  |
| Keeley (protocol available) | Schizophrenia & Bipolar | Purposive | Focus groups | Thematic iterative constant comparison, “grounded in the data”, Dual coding |
|  |  |  | Interviews |  |
|  |  |  | Delphi |  |
|  |  |  | Consensus meeting |  |
|  |  |  | Literature review |  |
|  |  |  | Stakeholder discussion |  |
|  |  |  | Survey |  |
| Maclennan *(Lam)* (protocol available) | Prostate cancer | Purposive | Systematic review | Thematic |
|  |  |  | Interviews |  |
|  |  |  | Focus groups |  |
|  |  |  | Delphi |  |
|  |  |  | Consensus meeting |  |
| Waters (protocol available) | Oropharyngeal Cancer | Maximum diversity sampling | Systematic review | Constant comparative, Dual coding |
|  |  |  | Interviews |  |
|  |  |  | Delphi |  |
|  |  |  | Consensus meeting |  |
| Alam | Facial aging | Not stated | Consensus conference | Not stated |
|  |  |  | Consensus meeting |  |
|  |  |  | Delphi process |  |
|  |  |  | Interview |  |
|  |  |  | Systematic review |  |
| Azuara-Blanco | Diabetic retinopathy | Not stated | Systematic review | Not stated |
|  |  |  | Delphi |  |
|  |  |  | Interviews |  |
|  |  |  | Focus groups |  |
|  |  |  | Survey |  |
| Azuara-Blanco | Age-related macular degeneration | Not stated | Systematic review | Not stated |
|  |  |  | Delphi |  |
|  |  |  | Interviews |  |
|  |  |  | Focus groups |  |
|  |  |  | Survey |  |
| Blazeby | Colorectal cancer | Not stated | Systematic review | Not stated |
|  |  |  | Interviews |  |
|  |  |  | Delphi |  |
|  |  |  | Consensus meeting |  |
| Cartee | Skin cancer excision | Not stated | Consensus conference | Not stated |
|  |  |  | Delphi process |  |
|  |  |  | Focus group(s) |  |
|  |  |  | Interview |  |
|  |  |  | Nominal group technique (NGT) |  |
|  |  |  | Systematic review |  |
| Chazapis | Anaesthesia | Not stated | Systematic review | Not stated |
|  |  |  | Survey |  |
|  |  |  | Interviews |  |
|  |  |  | Delphi |  |
| Clark | Acne scarring | Not stated | Consensus conference | Not stated |
|  |  |  | Delphi process |  |
|  |  |  | Focus group(s) |  |
|  |  |  | Interview |  |
|  |  |  | Nominal group technique (NGT) |  |
|  |  |  | Systematic review |  |
| Colavincenzo | Hair loss, non-scarring alopecia | Not stated | Consensus conference | Not stated |
|  |  |  | Delphi process |  |
|  |  |  | Focus group(s) |  |
|  |  |  | Interview |  |
|  |  |  | Nominal group technique (NGT) |  |
|  |  |  | Systematic review |  |
| Costa | Lower limb fractures | Not stated | Systematic review | Not stated |
|  |  |  | Interviews |  |
|  |  |  | Consensus meeting |  |
| Coulman | Obesity | Not stated | Systematic review | Not stated |
|  |  |  | Interviews |  |
|  |  |  | Delphi |  |
|  |  |  | Consensus meeting |  |
| Davidson | Liver | Not stated | Systematic review | Not stated |
|  |  |  | Interviews |  |
|  |  |  | Survey |  |
|  |  |  | Consensus meeting |  |
| Denniston | Uveitis | Not stated | Focus groups | Not stated |
|  |  |  | Interviews |  |
|  |  |  | Systematic review |  |
|  |  |  | Delphi |  |
|  |  |  | Consensus meeting |  |
| Duffy | Endometriosis | Not stated | Systematic review | Not stated |
|  |  |  | Focus groups |  |
|  |  |  | Delphi |  |
| Duncan-Millar | Upper limb rehabilitation after stroke | Not stated | Systematic review | Not stated |
|  |  |  | Focus groups |  |
|  |  |  | Interviews |  |
|  |  |  | Delphi |  |
|  |  |  | Survey |  |
| Fabricius | Prostate cancer | Not stated | Systematic review | Not stated |
|  |  |  | Interviews |  |
|  |  |  | Consensus meeting |  |
|  |  |  | Delphi |  |
|  |  |  | Consensus meeting |  |
| Forster | Rehabilitation (older people) | Not stated | Literature review | Grounded theory with constant comparison, Q-methodology |
|  |  |  | Interviews |  |
|  |  |  | Focus groups |  |
|  |  |  | Delphi |  |
| Goncalves | Dementia | Not stated | Clinical experts | Not stated |
|  |  |  | Consumers |  |
|  |  |  | Consumers |  |
|  |  |  | Families |  |
|  |  |  | Patient/support group representatives |  |
|  |  |  | Researchers |  |
| Iyengar | Rosacea | Not stated | Consensus conference | Not stated |
|  |  |  | Delphi process |  |
|  |  |  | Focus group(s) |  |
|  |  |  | Interview |  |
|  |  |  | Nominal group technique (NGT) |  |
|  |  |  | Systematic review |  |
| Kaufman | Childhood vaccination | Not stated | Systematic review | Not stated |
|  |  |  | Interviews |  |
|  |  |  | Delphi |  |
| Knight | Transplant surgery | Not stated | Delphi process | Not stated |
|  |  |  | Interview |  |
|  |  |  | Systematic review |  |
| Marson | Epilepsy | Not stated | Interviews | Thematic |
|  |  |  | Focus groups |  |
|  |  |  | Discrete choice experiments |  |
|  |  |  | Survey |  |
| Myles | Anaesthesia | Not stated | Systematic review | Not stated |
|  |  |  | Surveys |  |
|  |  |  | Interviews |  |
|  |  |  | Delphi |  |
| Reilly | Dementia | Not stated | Literature review | Not stated |
|  |  |  | Focus group |  |
|  |  |  | Delphi |  |
|  |  |  | Systematic review |  |
|  |  |  | Stated preference survey |  |
| Shokeen | Post Inflammatory Hyperpigmentation | Not stated | Consensus conference | Not stated |
|  |  |  | Delphi process |  |
|  |  |  | Focus group(s) |  |
|  |  |  | Interview |  |
|  |  |  | Nominal group technique (NGT) |  |
|  |  |  | Systematic review |  |
| Smith | Miscarriage | Not stated | Systematic review | Not stated |
|  |  |  | Interviews |  |
|  |  |  | Focus groups |  |
|  |  |  | Delphi |  |
|  |  |  | Consensus meeting |  |
| Tang | Melasma | Not stated | Consensus conference | Not stated |
|  |  |  | Delphi process |  |
|  |  |  | Focus groups |  |
|  |  |  | Interview |  |
|  |  |  | Nominal group technique (NGT) |  |
| Vasic | Leg veins | Not stated | Consensus conference | Not stated |
|  |  |  | Delphi process |  |
|  |  |  | Focus group(s) |  |
|  |  |  | Interview |  |
|  |  |  | Nominal group technique (NGT) |  |
|  |  |  | Systematic review |  |
| Vasic | Actinic keratosis | Not stated | Consensus conference | Not stated |
|  |  |  | Delphi process |  |
|  |  |  | Focus group(s) |  |
|  |  |  | Interview |  |
|  |  |  | Nominal group technique (NGT) |  |
|  |  |  | Systematic review |  |
| Whitehead | Cardiac arrest | Not stated | Systematic review | Not stated |
|  |  |  | Interviews |  |
|  |  |  | Focus groups |  |
|  |  |  | Delphi |  |
|  |  |  | Consensus meeting |  |
| Zajicek | Neurodegenerative diseases | Not stated | Systematic review | Not stated |
|  |  |  | Focus groups |  |
|  |  |  | Interviews |  |
|  |  |  | Survey |  |
| Zha | Scar | Not stated | Consensus conference | Not stated |
|  |  |  | Focus group(s) |  |
|  |  |  | Interview |  |
|  |  |  | Nominal group technique (NGT) |  |
|  |  |  | Systematic review |  |
| Zoet | Cardiovascular disease | Not stated | Systematic review | Not stated |
|  |  |  | Focus group |  |
|  |  |  | Delphi |  |
