# Supplementary material for: A review of patient and carer participation and the use of qualitative research in the development of core outcome sets
Source: PLoS One. 2017 Mar 16;12(3):e0172937. doi: 10.1371/journal.pone.0172937 (PMC5354261; doi:10.1371/journal.pone.0172937)
Supplement: S3 Appendix — (DOCX) [file pone.0172937.s003.docx]

**S3 Appendix – Unpublished work**

1. Jones JE. Core outcome set development: Understanding how qualitative research approaches can help to accommodate patient preferred outcomes in trial research: University of Birmingham; Ongoing PhD project.
